# Intrapleural treatment in patients with non‐small cell lung cancer with malignant pleural effusions in the real world

**DOI:** 10.1111/1759-7714.14224

**Published:** 2021-11-06

**Authors:** Pengfei Pan, Fengjuan Wu, Zhiyun Xu, Xiang Ji, Qian Qi, Xiaomin Huang, Ruyue Zhao, Mingtao Liu, Peng Jiang, Yu Li, Lisheng Xu

**Affiliations:** ^1^ Department of Pulmonary and Critical Care Medicine, Qilu Hospital, Cheeloo College of Medicine Shandong University Jinan China; ^2^ Department of Pulmonary and Critical Care Medicine Heze Municipal Hospital Heze China; ^3^ Department of Pulmonary and Critical Care Medicine Qilu Hospital of Shandong University Jinan China; ^4^ Department of Pulmonary and Critical Care Medicine Qianfoshan Hospital of Shandong First Medical University Jinan China; ^5^ Department of Pulmonary and Critical Care Medicine Binzhou People's Hospital Binzhou China; ^6^ Department of Pulmonary and Critical Care Medicine Weihai Municipal Hospital Weihai China

**Keywords:** carcinoembryonic antigen ratio, intrapleural therapy, lung cancer, overall survival, real world study

## Abstract

**Background:**

The aim of the study was to assess the efficacy and side‐effects of intrapleural treatment in non‐small cell lung cancer (NSCLC) patients with malignant pleural effusions (MPEs).

**Methods:**

The medical records of NSCLC patients with MPEs diagnosed in four Chinese hospitals from October 2014 to December 2019 were searched. The Kaplan–Meier method is used to calculate median overall survival (MOS) and subgroup analyses are done.

**Results:**

A total of 285 patients were evaluated; 81.1% of patients received intrapleural treatment, and no patients received talc pleurodesis. MOS of the whole cohort was 21 months. Patients were divided into three groups: erythromycin group (EG; intrapleural treatment with drugs and erythromycin); intrathoracic treatment group (ITG; intrapleural treatment with drugs); control group (CG; no drug treatment in the pleural cavity). The MOS of patients in the EG, ITG and CG was 20, 22, and 19 months, respectively. Among patients who received only chemotherapy as systemic therapy, the MOS of intrathoracic administration group (IAG; i.e., EG and ITG) was longer than that of CG (12 vs. 6 months; *p* = 0.034), and the MOS of patients with a ratio of carcinoembryonic antigen in pleural effusion (PE‐CEA): CEA in blood (B‐CEA) ≤1 is worse than that of patients with a ratio >1 (4 vs. 12 months, *p* = 0.021) and that of CG (4 vs. 6 months, *p* = 0.442).

**Conclusions:**

Intrapleural treatment can prolong the survival of NSCLC patients with MPE who do not receive targeted treatment or who only receive chemotherapy. The PE‐CEA: B‐CEA ratio can be used to predict the efficacy if intrapleural treatment is indicated.

## Graphical abstract

There is a debate about a malignant pleural effusion (MPE) necessitates intrapleural treatment. 285 non‐small cell lung cancer (NSCLC) patients with MPEs were included in the cohort. Among patients who received only chemotherapy as systemic therapy or who don't receive targeted treatment, the median overall survival of patients of intrathoracic administration group (IAG; intrapleural treatment with drugs) was longer than that of control group (CG; no drug treatment in the pleural cavity). Intrapleural treatment can prolong the survival of NSCLC patients with MPEs who don't receive targeted treatment or who receive only chemotherapy.

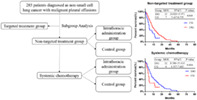

## INTRODUCTION

In China, lung cancer carries the highest morbidity and mortality of all cancer‐related diseases.^1^ The incidence of malignant pleural effusion (MPE) is between 7% and 23% in lung cancer.^2^ The accumulation of pleural effusion (PE) causes the patients to have symptoms such as cough, chest pain or dyspnea, which affects the quality of life of patients.^3^ At present, the goal of treatment of MPE is palliative. The purpose of treatment is to relieve symptoms and reduce the patient's hospital stay.^4^ Common treatment methods include catheter drainage and pleural fixation.^5^ Talc is the most effective pleural fixative.^4^ Because no medical talc powder that can be injected into the chest cavity has been produced and sold in China, some doctors may use drugs to carry out intrapleural treatment.

Although it is controversial whether a MPE necessitates intrapleural treatment, Zhong et al. found that the PE‐objective response rate (ORR) of MPE patients after intrapleural injection of nedaplatin was 62.73%, and the PE‐ORR of patients in the cisplatin group was 54.13%, while the PE‐ORR of patients in the lung cancer subgroup who received platinum‐based intrapleural treatment was 52.2%.^6^ A study conducted by Hong et al. showed that the PE‐ORR of patients with lung adenocarcinoma who received bevacizumab intrapleural treatment while receiving systemic chemotherapy followed by bevacizumab systemic treatment was 81%.^7^ These results indicate that intrathoracic treatment may be effective for MPE caused by lung cancer, but the study cohort is small and further research is needed. Miller and colleagues, after treating the pleural cavity of rabbits with erythromycin, discovered that the proportion of fibroblasts and endothelial cells in the subcutaneous tissue of the rabbit pleura was similar to that of the subcutaneous tissue after doxycycline treatment.^8^ Balassoulis et al. used erythromycin as a pleural sclerosing agent for recurrent pleural effusion and showed an PE‐ORR of 88.2%.^9^ These results indicate that erythromycin has the potential to become a pleural sclerosing agent.

Here, we retrospectively evaluated the efficacy and adverse reactions of different intrapleural treatments of non‐small cell lung cancer (NSCLC) patients with MPE in four hospitals in Shandong Province, China.

## METHODS

### Ethical approval of the study protocol

We collected relevant data from the electronic medical records of patients, and did not carry out any interventions. The Medical Ethics Committee permitted exemption from obtaining written informed consent (2019184) because this was a retrospective study.

### Patients

Using four hospital databases, we searched for patients discharged from October 2014 to December 2019 who were diagnosed with a PE and lung cancer. Patients were selected according to inclusion and exclusion criteria.

The inclusion criteria were: (i) an imaging diagnosis of a PE; (ii) pathological diagnosis of metastasis from NSCLC, and lung‐cancer cells in PEs; (iii) patients were receiving treatment for ≥2 cycles.

The exclusion criteria were: (i) no diagnosis of lung cancer; (ii) unknown disease; (iii) small‐cell lung cancer; (iv) participation in another clinical trial; (v) a PE caused by another disease; (vi) <2 cycles of treatment.

### Treatment and grouping

Patients were divided into three groups according to intrapleural treatment: erythromycin group (EG; intrapleural treatment with drugs and erythromycin); intrathoracic treatment group (ITG; intrapleural drug treatment); control group (CG; drugs not administered into the pleural cavity). Systemic treatment was based on National Comprehensive Cancer Network guidelines.^10^

### Patient characteristics

The date of the first diagnosis of MPE was documented as the baseline date. Patient characteristics at baseline were age, sex, smoking status, clinical symptoms, Eastern Cooperative Oncology Group performance status (ECOG PS) score, pathology, driver gene, clinical stage, atelectasis, blood level of carcinoembryonic antigen (B‐CEA), and CEA level in the PE (PE‐CEA). During follow‐up, information on the line of intrapleural treatment (first‐line treatment: intrapleural treatment within the fourth period of systemic therapy; second‐line treatment: intrapleural treatment after the fourth period of systemic therapy), local treatment regimen, and systemic treatment regimen was collected.

### Follow‐up

The follow‐up period ended in October 2020. Follow‐up evaluation comprised evaluation of the PE and solid tumor by computed tomography, which was carried out after every two cycles of therapy. According to a study by Nio and colleagues, evaluation of the response of a PE to therapy was divided into: complete response (CR: total disappearance of the PE for ≥4 weeks); partial response (PR: ≥50% reduction in the PE volume compared with the volume of the original PE for ≥4 weeks); stable disease (SD: <50% reduction in the PE volume and/or ≤25% increase in the PE volume compared with the volume of the original PE); progressive disease (PD: >25% increase in the volume of the original PE).^11^

The response to solid‐tumor treatment was evaluated with Response Evaluation Criteria for Solid Tumors 1.1. The classifications were: CR (disappearance of all target lesions, and pathological lymph nodes [target or nontarget] must have a reduction in the short axis to <10 mm); PR (≥30% decrease in the sum of the diameters of target lesions, using as reference the sum diameters at baseline); PD (≥20% increase in the sum of diameters of target lesions, taking as reference the smallest sum diameter in our study; an absolute increase of ≥5 mm or the appearance of one or more new lesions was also considered to denote progression); SD (neither sufficient shrinkage to qualify for PR nor sufficient increase to qualify for PD taking as reference the smallest sum diameter in our study).^12^

### Adverse events

Bone‐marrow toxicity and gastrointestinal reactions were graded according to Common Terminology Criteria for Adverse Events 4.0.^13^ Chest pain was classified as grade I to grade III according to the World Health Organization Verbal Rating Scale.^14^

### Endpoints

The primary endpoint was overall survival (OS), which was defined as the date from treatment initiation to death from any cause or the date of the last follow‐up. Secondary endpoints were the PE‐ORR and systemic objective remission rate (S‐ORR) at 6 weeks and 12 weeks. The ORR was calculated as the number of CR + PR patients/total number of patients (excluding those who withdrew from the study).

### Statistical analyses

First, the normality of baseline data was tested. Classified variables are expressed as numerical values and percentages. Continuous variables are expressed as the mean ± standard deviation. Continuous variables were compared by analysis of variance or Mann–Whitney U test. Classified variables were compared by the chi‐square test or Fisher's exact test. The survival time was drawn using the Kaplan–Meier method and compared with the log‐rank test, and subgroup analysis was carried out. The Cox proportional hazard regression model was employed to analyze the risk factors of OS and to calculate the hazard ratio. SPSS 26.0 (IBM) was employed for statistical analyses. *p* < 0.05 was considered statistically significant.

## RESULTS

### Baseline characteristics

Initially, the electronic medical records of 643 patients were retrieved. In reference to the inclusion and exclusion criteria, 285 patients were enrolled (Supplement Figure S1). The mean age of the three groups was ~60 years. The ITG and EG had more female patients than that in the CG (*p* = 0.025). The most common symptom of patients in the three groups was dyspnea. All patients had stage IV disease (according to the eighth edition of the American Joint Commission on Cancer TNM staging system for NSCLC). The percentage of patients accepting intrapleural treatment was 81.1%. The intrapleural treatment regimen of the EG was erythromycin combined with platinum. The intrapleural treatment regimen of the ITG comprised platinum, interleukin‐2, bevacizumab, and 5‐fluorouracil. Patients in the CG did not undergo treatment in the pleural cavity. The characteristics of the three groups of patients at baseline were comparable (Table 1).

**TABLE 1:** Baseline characteristics and treatment regimens

Characteristics	EG (*N* = 37)	ITG (*N* = 194)	CG (*N* = 54)	*p* value
Age (years)	60.84 ± 12.89	62.25 ± 11.25	59.78 ± 11.73	0.348
Sex				0.025
Male	23(62.2%)	89(45.9%)	18(33.3%)	
Female	14(37.8%)	105(54.1%)	36(66.7%)	
Smoking status				0.814
Current	14(37.8%)	65(33.5%)	20(37.0%)	
Occasionally/never	23(62.2%)	129(66.5%)	34(63.0%)	
Symptom*1				
Cough	23(62.2%)	103(53.1%)	31(57.4%)	
Dyspnea	25(67.6%)	141(72.7%)	40(74.1%)	
Chest pain	8(21.6%)	41(21.1%)	14(25.9%)	
Others	1(2.7%)	2(1%)	1(1.9%)	
None	1(2.7%)	8(4.1%)	1(1.9%)	
ECOG PS scores				0.147
0–1	29(78.4%)	168(86.6%)	50(92.6%)	
2	8(21.6%)	26(13.4%)	4(7.4%)	
Pathology				0.110
Adenocarcinoma	32(86.5%)	185(95.4%)	50(92.6%)	
Squamous cell carcinoma /other	5(13.5%)	9(4.6%)	4(7.4%)	
Driver gene				0.879
Positive	15(57.7%)	68(63.0%)	17(63.0%)	
Negative	11(42.3%)	40(37.0%)	10(37.0%)	
Missing	11(0.0%)	86(0.0%)	27(0.0%)	
Blood CEA (ng/ml)				0.056
≥10	27(75.0%)	107(59.8%)	23(48.9%)	
<10	9(25.0%)	72(40.2%)	24(51.1%)	
Missing	1(0.0%)	15(0.0%)	7(0.0%)	
PE‐CEA: B‐CEA				0.840
>1	22(95.7%)	86(89.6%)	26(89.7%)	
≤1	1(4.3%)	10(10.4%)	3(10.3%)	
Missing	14(0.0%)	98(0.0%)	25(0.0%)	
Atelectasis				0.196
Yes	4(13.3%)	28(15.3%)	13(25.5%)	
No	26(86.7%)	155(84.7%)	38(74.5%)	
Missing	7(0.0%)	11(0.0%)	3(0.0%)	
Intrapleural treatment line*2				0.795
First‐line	31(83.8%)	167(86.5%)		
Second‐line	6(16.2%)	26(13.5%)		
Intrathoracic treatment regimen				
Erythromycin plus chemotherapy*3	37			
Chemotherapy*4		150		
Chemotherapy plus other drugs*5		29		
Immunomodulatory therapy*6		9		
Antiangiogenic therapy*7		6		
No treatment/only pumping			54	
Systemic treatment regimen				0.132
Chemotherapy	18(48.6%)	70(36.1%)	18(33.3%)	
Targeted therapy*8	14(37.8%)	103(53.1%)	31(57.4%)	
Antiangiogenic therapy*9	2(5.4%)	17(8.8%)	3(5.6%)	
Immunotherapy*10	1(2.7%)	3(1.5%)	0(0%)	
No treatment	2(5.4%)	1(0.5%)	2(3.7%)	
Local treatment regimen				0.098
Radiotherapy/PCI	1(2.7%)	19(9.8%)	6(11.1%)	
Radiofrequency ablation*11	1(2.7%)	0(0%)	1(1.9%)	
Surgery*12	1(2.7%)	1(0.5%)	0(0%)	
No treatment	34(91.9%)	174(89.7%)	47(87.0%)	

*Note*: *1, symptom ratio = number of symptomatic patients/number in each group; *2, first‐line: intrapleural treatment within the fourth period of systemic treatment; second‐line: intrapleural treatment after the fourth period of systemic treatment; *3, 37 patients accepted platinum; *4, 149 patients accept platinum and one patient accepted 5‐fluorouracil; *5, 25 patients accepted platinum and immunomodulatory drugs (interleukin, staphylococcal enterotoxin C) and four patients accepted platinum and bevacizumab; *6, interleukin or staphylococcal enterotoxin C; *7, bevacizumab; *8, 85 patients accepted targeted therapy and chemotherapy. 35 patients accepted targeted therapy and chemotherapy and antiangiogenic therapy. 24 patients accepted targeted therapy alone. Three patients accepted targeted therapy and antiangiogenic therapy. One patient accept targeted therapy, chemotherapy, antiangiogenic therapy and immunotherapy; *9, 21 patients accepted antiangiogenic therapy and chemotherapy and one patient accept antiangiogenic therapy alone; *10, Three patients accepted immunotherapy, chemotherapy, and antiangiogenic therapy, and one patient accepted immunotherapy combined with chemotherapy; *11, radiofrequency ablation combined with radiotherapy was carried out in two patients; *12, Two patients accepted surgery and radiotherapy.

Abbreviations: CEA, carcinoembryonic antigen; CG, control group; ECOG PS scores, Eastern Cooperative Oncology Group performance status scores; EG, erythromycin group; ITG, intrathoracic treatment group; PCI, prophylactic cranial irradiation; PE‐CEA: B‐CEA, CEA in pleural effusion: CEA in blood.

### Study endpoints

**FIGURE 1:**
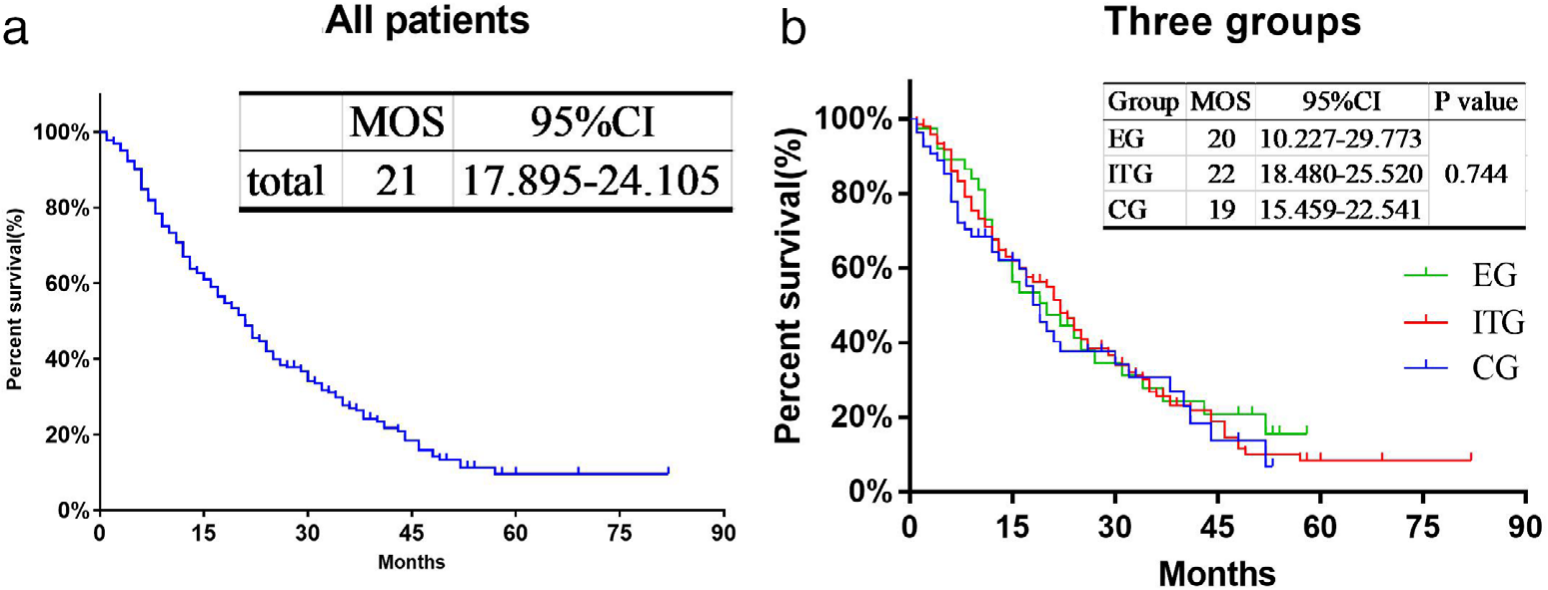
Analysis of all patients. (a) Kaplan–Meier curves of overall survival of all patients. (b) Kaplan–Meier curves of overall survival of patients in the three groups; CG, control group; CI, confidence interval; EG, erythromycin group; ITG, intrathoracic treatment group; MOS, median overall survival [Correction added on 11 November 2021, after first online publication: figure 1 has been updated.]

The median duration of follow‐up of all patients was 16 months. The median overall survival (MOS) of all patients was 21 (95% CI: 17.895–24.105) months. The MOS of patients in the EG, ITG and CG was 20, 22, and 19 months, respectively (Figure 1). There was no significant difference in MOS within the three groups or between the three groups in the pairwise comparison.

In the comparison of short‐term efficacy, at 6 and 12 weeks, the PE‐ORR and S‐ORR of the EG were superior to those of the ITG and CG, but the difference in the three groups was not significant (Table 2).

**TABLE 2:** ORR of pleural effusion and system

Pleural effusion	EG (*N* = 37)	ITG (*N* = 194)	CG (*N* = 54)	*p*‐value
6‐week PE‐ORR	75.8%	62.4%	71.7%	0.217
12‐week PE‐ORR	76.7%	70.5%	72.7%	0.776
System				
6‐week S‐ORR	44.4%	43.3%	47.7%	0.868
12‐week S‐ORR	50.0%	36.7%	46.3%	0.264

*Note*: The ORR was calculated as the number of CR + PR patients/total number of patients (excluding those who withdrew from the study).

Abbreviations: CG, control group; EG, erythromycin group; ITG, intrathoracic treatment group; PE‐ORR, pleural effusion objective remission rate; S‐ORR, systemic objective remission rate.

### Subgroup analyses

#### Regimen for systemic treatment

According to the regimen for systemic treatment, patients were divided into a targeted treatment group and nontargeted treatment group. The MOS of patients in the targeted treatment group was significantly longer than that of patients in the nontargeted treatment group (32 vs. 12 months, *p* < 0.0001). At baseline, the characteristics of the three groups in the targeted treatment group and nontargeted treatment group were comparable, and there was no significant difference in the systemic treatment regimen (Supplement S1–S5).

**FIGURE 2:**
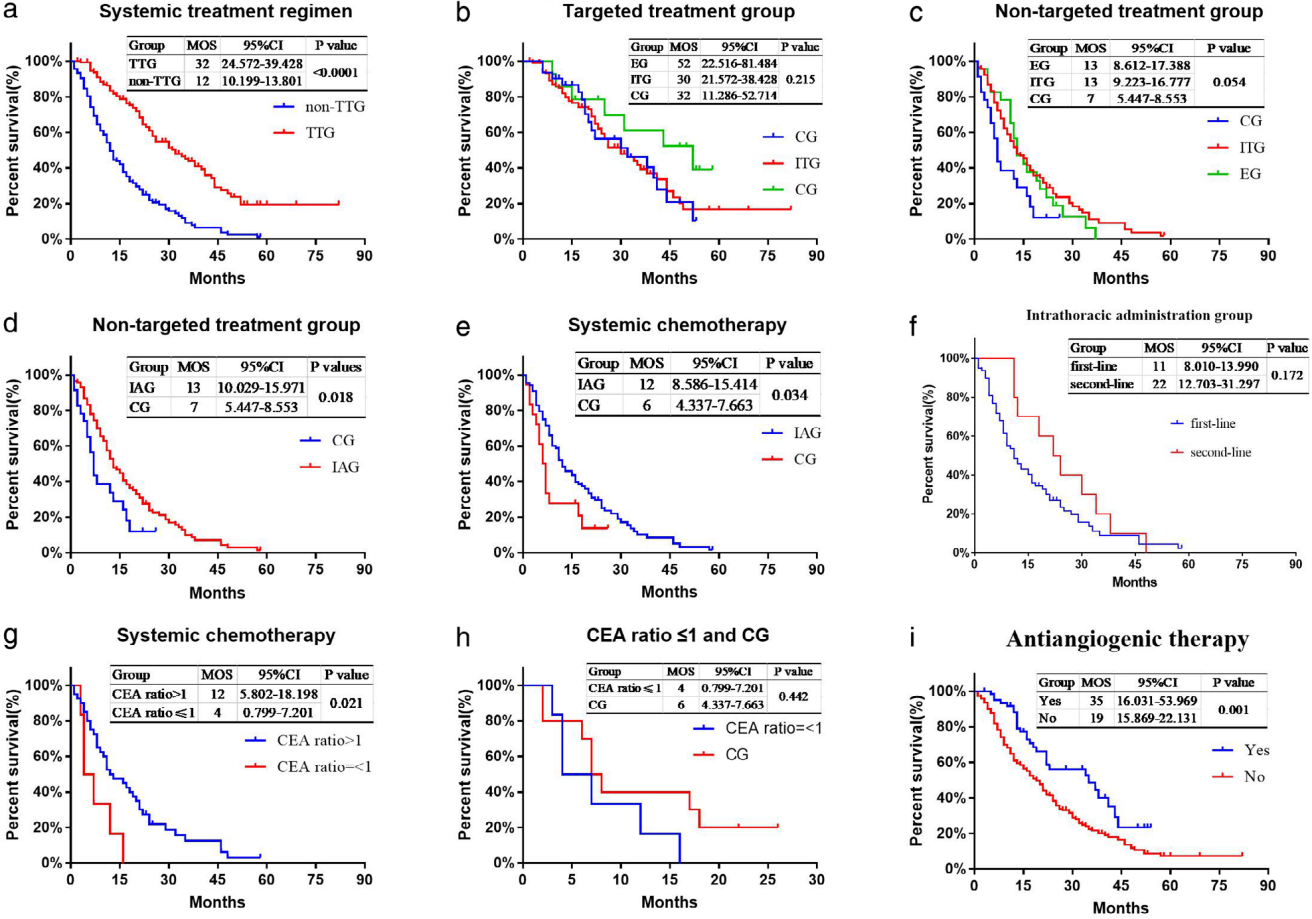
Subgroup analysis. (a) Kaplan–Meier curves of overall survival of patients accepting systemic treatment regimen in the targeted and nontargeted treatment groups. (b) Kaplan–Meier curves of overall survival of patients of three groups in the targeted treatment group. (c) Kaplan–Meier curves of overall survival of patients of three groups in the nontargeted treatment group. (d) Kaplan–Meier curves of overall survival of patients accepting nontargeted treatment in the control and intrathoracic administration groups. (e) Kaplan–Meier curves of overall survival of patients accepting systemic chemotherapy in the control and intrathoracic administration groups. (f) Kaplan–Meier curves of overall survival of patients accepting first‐ or second‐line intrapleural treatment in the intrathoracic treatment group. (g) Kaplan–Meier curves of overall survival of patients accepting systemic chemotherapy in the PE‐CEA:B‐CEA > 1 group and PE‐CEA:B‐CEA ≤ 1 group. (h) Kaplan–Meier curves of overall survival of patients accepting systemic chemotherapy in the CEA ratio > 1 group and control group. (i) Kaplan–Meier curves of overall survival of patients accepting antiangiogenic therapy who did not accept antiangiogenic therapy; CEA, carcinoembryonic antigen; CG, control group; CI, confidence interval; EG, erythromycin group; IAG, intrathoracic administration group; ITG, intrathoracic treatment group; MOS, median overall survival; non‐TTG, nontargeted treatment group; TTG, targeted treatment group

There were no significant differences between the three groups of patients in the pairwise comparison in the targeted treatment group. However, patients in the ITG had longer MOS than that of patients in the CG in the nontargeted treatment group (13 vs. 7 months, *p* = 0.022). Patients in the EG had longer MOS than that of patients in the CG in the nontargeted treatment group (13 vs. 7 months, *p* = 0.084). A significant difference between the ITG and EG for MOS was not observed (*p* = 0.663). (Figure 2).

The intrapleural administration group (IAG) comprised the EG and ITG. Patients in the IAG did not benefit from longer MOS than patients in the CG in the targeted treatment group (31 vs. 32 months, *p* = 0.660). The MOS of patients in the IAG was 6‐months longer than that of the CG in the nontargeted treatment group (13 vs. 7 months, *p* = 0.018). Among patients who accepted chemotherapy only as systemic treatment, the MOS of the IAG was significantly longer than that of the CG (12 vs. 6 months, *p* = 0.034) (Figure 2).

#### Line of intrapleural treatment

The MOS of patients undergoing first‐line intrapleural treatment was shorter than that of patients undergoing second‐line intrapleural treatment, but the difference was not significant (20 vs. 30 months, *p* = 0.198). There was no significant difference in the MOS of first‐line‐treatment patients and that of second‐line‐treatment patients in the target treatment group and nontarget treatment group in subgroup analyses (31 vs. 34 months, *p* = 0.719; 12 vs. 22 months, *p* = 0.087). Among patients who accepted only chemotherapy as systemic treatment, second‐line treatment patients survived longer than first‐line‐treatment patients, but the difference was not significant (22 vs. 11 months, *p* = 0.172).

#### PE‐CEA: B‐CEA ratio

Patients who had a ratio of PE‐CEA: B‐CEA > 1 had a longer MOS than that of patients with a ratio of PE‐CEA: B‐CEA ≤1 (22 m vs. 11 months, *p* = 0.034). There was no significant difference between these two groups of patients in the targeted treatment group (36 vs. 21 months, *p* = 0.510), but a significant difference was noted between these two groups of cases in the nontargeted treatment group (13 vs. 4 months, *p* = 0.015). Among patients who accepted only chemotherapy as systemic treatment, patients who had a ratio of PE‐CEA: B‐CEA > 1 survived longer than patients with a ratio of PE‐CEA: B‐CEA ≤1 (12 vs. 4 months, *p* = 0.021), but the MOS of patients with a ratio of PE‐CEA: B‐CEA ≤1 was shorter than that of patients in the CG (4 vs. 6 months, *p* = 0.442) (Figure 2).

#### Antiangiogenic therapy

Sixty‐three patients received antiangiogenic therapy, but the remainder of patients did not. The MOS of patients who received antiangiogenic therapy was significantly longer than that of patients who did not receive antiangiogenic therapy (35 vs. 19 months, *p* = 0.001). (Figure 2).

### Prognostic factors

**FIGURE 3:**
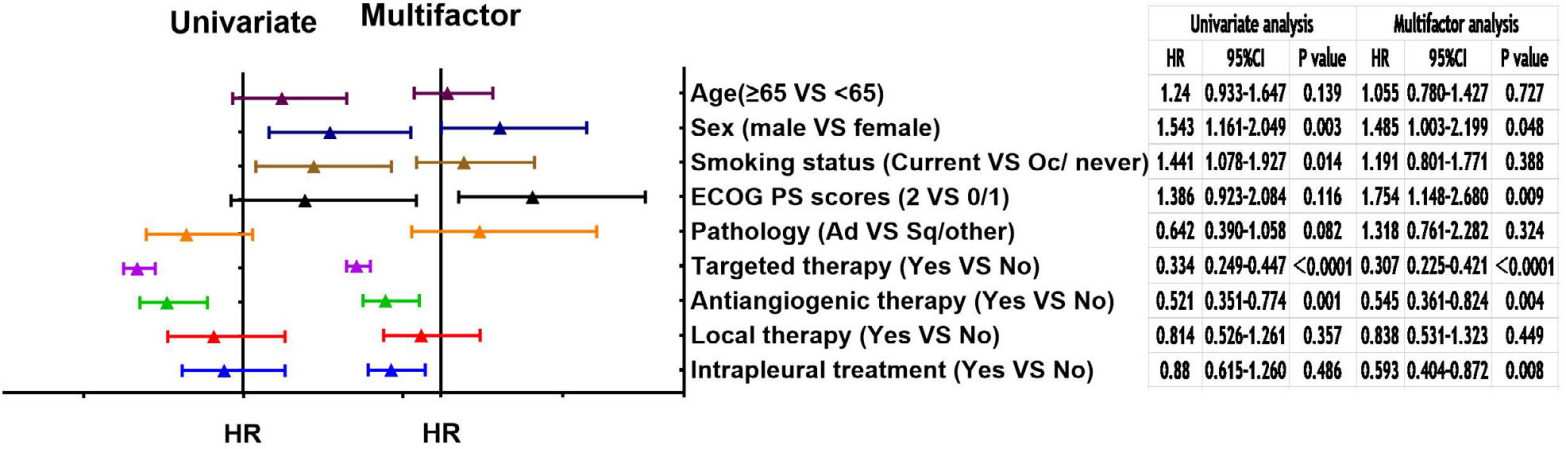
The hazard ratio of the risk factors of overall survival of all patients analyzed by Cox proportional hazard regression model. Ad, adenocarcinoma; CI, confidence interval; ECOG PS scores, Eastern Cooperative Oncology Group PS scores; HR, hazard ratio; Oc, occasionally; Sq, squamous cell carcinoma

The continuous variables and classification variables were convert into binary variable. Sex and ECOG PS score, targeted therapy, antiangiogenic therapy and intrapleural treatment affected the prognosis of patients (Figure 3).

### Adverse reactions

We documented the type and prevalence of adverse reactions in the three groups. The prevalence of chest pain in the EG was higher than that in the other two groups, but chest pain was the primary adverse event. There was no significant difference in the prevalence of bone‐marrow toxicity or gastrointestinal reactions among the three groups. Infection was not documented in any patient (Table 3).

**TABLE 3:** Adverse reactions

Adverse reactions	EG (*N* = 37)	ITG (*N* = 194)	CG (*N* = 54)	*p*‐value
Bone marrow toxicity				
Leukopenia				0.863
Grade I	13(35.1%)	50(25.8%)	15(27.8%)	
Grade II	5(13.5%)	23(11.9%)	8(14.8%)	
Grade III	4(10.8%)	21(10.8%)	4(7.4%)	
Neutropenia				0.973
Grade I	11(29.7%)	47(24.2%)	14(25.9%)	
Grade II	5(13.5%)	22(11.3%)	6(11.1%)	
Grade III	3(8.1%)	21(10.8%)	4(7.4%)	
Thrombocytopenia				0.601
Grade I	4(10.8%)	42(21.6%)	10(18.5%)	
Grade II	1(2.7%)	6(3.1%)	2(3.7%)	
Anemia				0.138
Grade I	6(16.2%)	53(27.3%)	7(13.0%)	
Grade II	3(8.1%)	11(5.7%)	5(9.3%)	
Gastrointestinal reaction				0.985
Grade I	13(35.1%)	72(37.1%)	17(31.5%)	
Grade II	4(10.8%)	20(10.3%)	5(9.3%)	
Grade III	4(10.8%)	17(8.8%)	5(9.3%)	
Chest pain				<0.0001
Grade I	14(37.8%)	4(2.1%)	1(1.9%)	
Grade II	1(2.7%)	5(2.6%)	2(3.7%)	
Grade III	2(5.4%)	0(0%)	0(0%)	

*Note*: Bone marrow toxicity and gastrointestinal reactions were graded according to the 4.0 version of Common Terminology Criteria for Adverse Events (CTCAE). According to the World Health Organization verbal rating scales (VRS), chest pain was classified as grade I–III.

Abbreviations: CG, control group; EG, erythromycin group; ITG, intrathoracic treatment group.

## DISCUSSION

Malignant pleural effusion occurs in 15% of cancer patients,^15^ including more than half of NSCLC patients.^16^ MPE is an independent risk factor for the prognosis of cancer patients and an important indicator of the staging of lung cancer patients.^17^ At present, local treatment is still the main treatment for MPE, including long‐term thoracic drainage, pleurodesis, pleurectomy and intrathoracic drug therapy.^4^, ^5^ However, not all patients need to receive intrapleural interventional therapy. International guidelines recommend that asymptomatic MPE patients should not undergo intrapleural treatment, and that symptomatic MPE patients with atelectasis should have an indwelling pleural catheter. Symptomatic patients with expandable lungs should undergo talc pleurodesis.^4^, ^5^ The percentage of patients undergoing intrapleural treatment in the present study was 81.1%, of which nine patients were asymptomatic and, among 45 patients with atelectasis, 32 patients received intrapleural treatment. No patients in our study underwent talc pleurodesis because only talc powder for external use, not talc powder for internal use, can be produced and sold in China. The reason for this dilemma may be that talc powder is not a high‐tech product, and the profit for production and distribution are very limited, and no one is willing to operate at a loss. All patients preferred to have tube drainage of their PE at hospital rather than at home. Our results suggested that the treatment of NSCLC patients with MPE in China was not identical to that recommended in international guidelines.

Previous studies state that the MOS of MPE patients is 3–12 months,^18^ and the MOS of MPE caused by lung cancer was 5.49 months.^19^ The MOS of all patients in our study was 21 months. The possible reasons for this are that all of our patients had NSCLC; 148 (51.9%) patients received targeted therapy.^10^, ^20^ We previously used platinum and erythromycin to treat NSCLC patients with MPE. The short‐term ORR of MPE was 81.8%, which was consistent with the PE‐ORR of EG.^21^ Animal experiments have shown that animals have pleural fibrosis after intrapleural injection of erythromycin, and clinical experiments have also shown that erythromycin is effective as a sclerosing agent for pleural adhesions.^8^, ^9^ In this study, the PE‐ORR and S‐ORR of EG are better than ITG (Appendix Table S5), which shows that erythromycin does help to control PE.

After the drug is injected into the pleural cavity, it can directly kill pleural tumor cells and stimulate inflammation of the pleura to reduce pleural fluid.^22^ At present, many intrapleural therapeutic drugs have been studied, such as cisplatin, bevacizumab, etc. A previous study showed that the PE‐ORR of lung cancer patients with MPE after intrathoracic injection of nedaplatin or cisplatin was more than 50%,^6^ and two studies of intrapleural injection of bevacizumab showed that compared to the control group, patients in the bevacizumab group had a higher MPE response rate.^7^, ^23^ In order to control the influence of systemic and local treatment regimen, we classified the treatment regimen of patients according to different drugs and analyzed the difference in distribution. The results showed that there was no statistical difference in the systemic and system treatment regimen of each group. We analyzed the MOS of cases in the EG and ITG and compared it with that of people in the CG. No survival benefit was observed in the targeted treatment group, considering the interference of targeted drugs.^10^, ^20^ In the nontargeted treatment group, the MOS of the IAG patients was 13 months, which was roughly the same as the data report by Du et al. (the MOS of the bevacizumab and cisplatin group was 10.3 months; the MOS of the cisplatin group was 10.1 months),^23^ significantly better than that of CG group. Among patients who received chemotherapy only as systemic therapy, the MOS of IAG patients was better than the data of Yoshida et al. (the MOS of the cisplatin and etoposide group was 45.7 weeks),^24^ which was twice that of CG patients. The reason for this result may be 40.1% of patients in the cisplatin and etoposide group in the study by Yoshida et al. did not accept systemic treatment. Our results suggests that intrapleural treatment can prolong the survival of patients who do not receive targeted treatment or who receive only chemotherapy.

Patients with lung cancer usually receive 4–6 cycles of chemotherapy.^10^ The line of intrapleural treatment is divided into first‐ and second‐line treatment depending on whether it is before or after the fourth cycle of systemic treatment, respectively, and indicates if patients accepted intrapleural treatment at an early or late stage. We showed that the MOS of patients who had undergone first‐line intrapleural treatment was shorter than that of patients who had undergone second‐line treatment, but the difference was not significant. This result may have been because the number of patients who underwent second‐line treatment was low.

Lung cancer tissue releases CEA. The latter is a polysaccharide–protein complex of molecular weight 22 kDa, so, in general, it cannot penetrate the pleura.^25^ Therefore, the increase in CEA level in PEs is often considered to be due to metastatic cells. We quantitatively expressed the distribution of tumor load by the ratio of PE‐CEA: B‐CEA. A PE‐CEA: B‐CEA ratio > 1 indicates that the tumor load is distributed mainly in the pleura. A PE‐CEA: B‐CEA ratio ≤ 1 indicates that the tumor load is distributed mainly in the whole body. We showed that, compared to patients with a PE‐CEA: B‐CEA ratio ≤ 1, the MOS of patients with a PE‐CEA: B‐CEA ratio > 1 increased considerably, whereas the MOS of patients with a PE‐CEA: B‐CEA ratio ≤ 1 was shorter than that of cases in the CG. These data suggest that not all NSCLC patients with MPE should undergo intrapleural treatment, and that the PE‐CEA: B‐CEA ratio may be an effective indicator of intrapleural treatment.

Previous studies showed that pathology, TNM stage and ECOG PS scores are accepted prognostic factors in MPE,^4^ and pH of the PE,^26^ level of lactate dehydrogenase,^27^ neutrophil lymphocyte ratio^28^ and serum level of albumin^29^ are potential prognostic factors. We showed that in addition to common prognostic factors such as ECOG PS scores, female and patients who received targeted therapy, antiangiogenic therapy or intrapleural therapy carried a lower risk of death.

To achieve higher drug concentration in the pleural cavity and lower drug toxicity in the whole body is the main goal of intrapleural treatment. Previous studies have shown that intravenous injection of cisplatin (100 mg/m^2^) can lead to a peak blood concentration of about 6 μg/ml,^30^ while Sakaguchi et al. perfused cisplatin (80 mg/m^2^) within the pleural cavity using hyperthermic‐chemotherapy principles, and the maximum concentration of platinum in blood was 0.66 ± 0.31 μg/ml.^31^ In our previous studies, after intravenous injection of lobaplatin (50 mg), the platinum concentration (in μg/ml) in the pleural cavity and plasma was 13.763 ± 1.523 and 1.120 ± 0.164, respectively.^21^ These results suggest that if the drug is injected into the pleural cavity, the proportion of drug in blood will be low. In our study, the incidence of chest pain in the EG was higher than that of the ITG and CG, but chest pain was the main adverse reaction, and its incidence was consistent with that in our previous study (35.7%).^21^ There was no significant difference in the incidence of gastrointestinal reactions in patients in the EG, ITG, or CG. These data suggest that intrapleural treatment did not increase the incidence of systemic adverse reactions in patients. The reason is that the pleural cavity injection does not increase the patient's blood concentration, and the adverse reactions are mainly caused by systemic chemotherapy. No infection occurred in any of our patients, which may have been because all patients underwent short‐term chest drainage.

Our study had two main limitations. First, this was a retrospective study and some information was lacking, which affected the data quality to a certain extent. Second, although we collected many variables of patients, some unmeasured factors may have confounded our analyses.

In conclusion, in China, the real‐world treatment of NSCLC patients with MPE is very different from that recommended in guidelines, mainly due to a large proportion of patients receiving intrapleural treatment. The latter can prolong the survival of NSCLC patients with MPE who do not receive targeted therapy or who receive only systemic chemotherapy. Not all NSCLC patients with MPE benefit from intrapleural treatment. The PE‐CEA: B‐CEA ratio can be used to predict the efficacy if intrapleural treatment is indicated.

## CONFLICT OF INTEREST

The authors declare that there are no competing interests.

## Supporting information


**Appendix S1**. Supporting Information
