# Supplementary material for: Intrapleural treatment in patients with non‐small cell lung cancer with malignant pleural effusions in the real world
Source: Thorac Cancer. 2021 Nov 6;12(24):3416–25. doi: 10.1111/1759-7714.14224 (PMC8671907; doi:10.1111/1759-7714.14224)
Supplement: Supplementary file 1 — Appendix S1. Supporting Information [file TCA-12-3416-s001.docx]

**Supplement**

**Figure 1 Study flow diagram;**


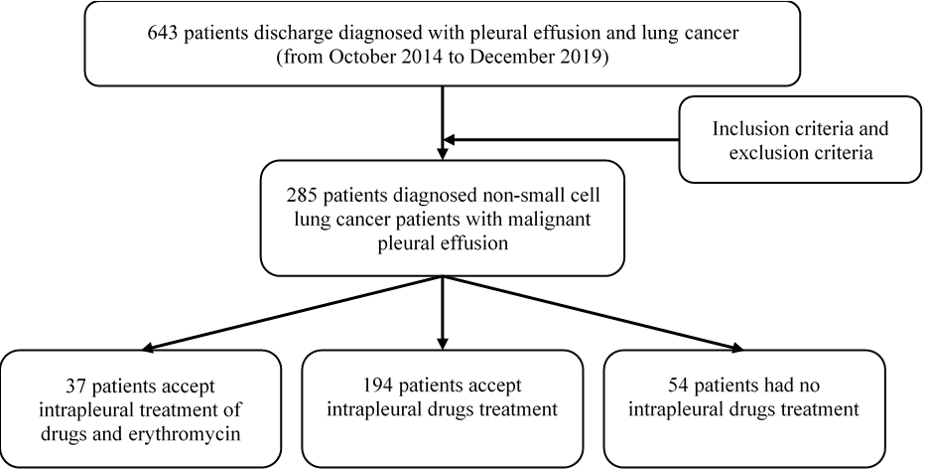


**Table 1 Baseline characteristics of targeted treatment group**

| Characteristics | EG(N=14) | ITG(N=103) | CG(N=31) | P value |
| --- | --- | --- | --- | --- |
| Age(years) | 62.07±9.41 | 61.31±11.98 | 59.39±11.75 | 0.679 |
| Sex |  |  |  | 0.481 |
| male | 7(50.0%) | 43(41.7%) | 10(32.3%) |  |
| female | 7(50.0%) | 60(58.3%) | 21(67.7%) |  |
| Smoking status |  |  |  | 0.667 |
| Current | 5(35.7%) | 29(28.2%) | 11(35.5%) |  |
| Occasionally/ never | 9(64.3%) | 74(71.8%) | 20(64.5%) |  |
| Symptom ^a^ |  |  |  |  |
| Cough | 8(57.14%) | 58(56.31%) | 19(61.29%) |  |
| Dyspnea | 8(57.14%) | 70(67.96%) | 24(77.42%) |  |
| Chest pain | 2(14.29%) | 23(22.33%) | 8(25.81%) |  |
| Others | 0(0.0%) | 1(0.97%) | 1(3.23%) |  |
| none | 1(7.14%) | 6(5.83%) | 1(3.23%) |  |
| ECOG PS scores |  |  |  | 0.304 |
| 0-1 | 11(78.6%) | 90(84.7%) | 29(93.5%) |  |
| 2 | 3(21.4%) | 13(12.6%) | 2(6.5%) |  |
| Pathology |  |  |  | 0.351 |
| Adenocarcinoma | 14(100.0%) | 101(98.1%) | 29(93.5%) |  |
| Squamous cell carcinoma / other | 0(0.0%) | 2(1.9%) | 2(6.5%) |  |
| Driver gene |  |  |  | 0.912 |
| Positive | 8(80.0%) | 61(83.6%) | 15(83.3%) |  |
| Negative | 2(20.0%) | 12(16.4%) | 3(16.7%) |  |
| missing | 4(0.0%) | 30(0.0%) | 13(0.0%) |  |
| Blood CEA (ng/ml) |  |  |  | 0.375 |
| ≥10 | 10(76.9%) | 55(59.8%) | 14(53.8%) |  |
| ＜10 | 3(23.1%) | 37(40.2%) | 12(46.2%) |  |
| missing | 1(0%) | 11(0.0%) | 5(0.0%) |  |
| PE-CEA: B-CEA |  |  |  | 0.471 |
| ＞1 | 9(100.0%) | 48(90.6%) | 18(100.0%) |  |
| ≤1 | 0(0.0%) | 5(9.4%) | 0(0.0%) |  |
| missing | 5(0.0%) | 50(0.0%) | 13(0.0%) |  |
| Atelectasis |  |  |  | 0.242 |
| Yes | 0(0.0%) | 14(14.1%) | 6(20.7%) |  |
| No | 12(100.0%) | 85(85.9%) | 23(79.3%) |  |
| missing | 2(0.0%) | 4(0.0%) | 2(0.0%) |  |
| Intrapleural treatment line ^b^ |  |  |  | 0.215 |
| First-line | 14(100.0%) | 86(83.5%) |  |  |
| Second-line | 0(26.1%) | 17(16.5%) |  |  |

a: symptom ratio = number of patients with symptoms / number of cases per group; b: first line (intrathoracic treatment within 4 cycles from systemic treatment) and second line (> 4 cycles intrathoracic treatment); Abbreviations: ECOG PS scores, Eastern Cooperative Oncology Group performance status scores; CEA: carcinoembryonic antigen; p/b CEA ratio, pleural effusion CEA to blood CEA ratio; EG, erythromycin group; ITG, intrathoracic treatment group; CG, control group;

**Table 2 Systemic treatment regimen of targeted treatment group**

| Treatment regimen | EG(N=14) | ITG(N=103) | CG(N=31) | Total |
| --- | --- | --- | --- | --- |
| Systemic treatment regimen |  |  |  |  |
| Targeted therapy | 14 | 103 | 31 | 148 |
| Local treatment regimen |  |  |  |  |
| Radiotherapy /PCI | 0 | 8 | 3 | 11 |

Abbreviations: EG, erythromycin group; ITG, intrathoracic treatment group; CG, control group; PCI, prophylactic cranial irradiation;

**Table 3 Baseline characteristics of non-targeted treatment group**

| Characteristics | EG(N=23) | ITG(N=91) | CG(N=23) | P value |
| --- | --- | --- | --- | --- |
| Age(years) | 60.09±14.77 | 63.32±10.31 | 60.30±11.94 | 0.322 |
| Sex |  |  |  | 0.061 |
| male  female | 16(69.6%)  7(30.4%) | 46(50.5%)  45(49.5%) | 8(34.8%)  15(65.2%) |  |
| Smoking status |  |  |  | 0.999 |
| Current | 9(39.1%) | 36(39.6%) | 9(39.1%) |  |
| Occasionally/ never | 14(60.9%) | 55(60.4%) | 14(60.9%) |  |
|  |  |  |  |  |
| Symptom ^a^ |  |  |  |  |
| Cough | 15(65.2%) | 45(49.5%) | 12(52.2%) |  |
| Dyspnea | 17(73.9%) | 71(78.0%) | 16(69.6%) |  |
| Chest pain | 6(26.1%) | 18(19.8%) | 6(26.1%) |  |
| Others | 1(4.3%) | 1(1.1%) | 0(0%) |  |
| none | 0(0%) | 2(2.2%) | 0(0%) |  |
| ECOG PS scores |  |  |  | 0.474 |
| 0-1 | 18(78.3%) | 78(85.7%) | 21(91.3%) |  |
| 2 | 5(21.7%) | 13(14.3%) | 2(8.7%) |  |
| Pathology |  |  |  | 0.173 |
| Adenocarcinoma | 18(78.3%) | 84(92.3%) | 21(91.3%) |  |
| Squamous cell carcinoma / other | 5(21.7%) | 7(7.7%) | 2(8.7%) |  |
| Driver gene |  |  |  | 0.203 |
| Positive  Negative | 7(43.8%)  9(56.3%) | 7(20.0%)  28(80.0%) | 2(22.2%)  7(77.8%) |  |
|  |  |  |  |  |
| missing | 7(0.0%) | 56(0.0%) | 14(0.0%) |  |
|  |  |  |  |  |
|  |  |  |  |  |
| Blood CEA (ng/ml) |  |  |  | 0.111 |
| ≥10 | 17(73.9%) | 52(59.8%) | 9(42.9%) |  |
| ＜10 | 6(26.1%) | 35(40.2%) | 12(57.1%) |  |
| missing | 0(0%) | 4(0.0%) | 2(0.0%) |  |
| PE-CEA: B-CEA |  |  |  | 0.397 |
| ＞1 | 13(92.9%) | 38(88.4%) | 8(72.7%) |  |
| ≤1 | 1(7.1%) | 5(11.6%) | 3(27.3%) |  |
| missing | 9(0.0%) | 48(0.0%) | 12(0.0%) |  |
| Atelectasis |  |  |  | 0.256 |
| Yes | 4(22.2%) | 14(16.7%) | 7(31.8%) |  |
| No | 14(77.8%) | 70(83.3%) | 15(68.2%) |  |
| missing | 5(0.0%) | 7(0.0%) | 1(0.0%) |  |
| Intrapleural treatment line ^b^ |  |  |  | 0.088 |
| First-line | 17(73.9%%) | 82(90.1%) |  |  |
| Second-line | 6(26.1%) | 9(9.9%) |  |  |

a: symptom ratio = number of patients with symptoms / number of cases per group; b: first line (intrathoracic treatment within 4 cycles from systemic treatment) and second line (> 4 cycles intrathoracic treatment); Abbreviations: ECOG PS scores, Eastern Cooperative Oncology Group performance status scores; CEA: carcinoembryonic antigen; PE-CEA: B-CEA, CEA in pleural effusion: CEA in blood; EG, erythromycin group; ITG, intrathoracic treatment group; CG, control group;

**Table 4 Systemic treatment regimen of non-targeted treatment group**

| Treatment regimen | EG(N=23) | ITG(N=91) | CG(N=23) | P value |
| --- | --- | --- | --- | --- |
| Systemic treatment regimen |  |  |  | 0.222 |
| Chemotherapy | 18(78.3%) | 70(76.9%) | 18(78.3%) |  |
| Antiangiogenic therapy ^a^ | 2(8.7%) | 17(18.7%) | 3(13.0%) |  |
| Immunotherapy ^b^ | 1(4.3%) | 3(3.3%) | 0(0%) |  |
| No treatment | 2(8.7%) | 1(1.1%) | 2(8.7%) |  |
| Local treatment regimen |  |  |  | 0.208 |
| Radiotherapy /PCI | 1(4.3%) | 11(12.1%) | 3(13.0%) |  |
| Radiofrequency ablation ^c^ | 1(4.3%) | 0(0%) | 1(4.3%) |  |
|  |  |  |  |  |
| Surgery ^d^ | 1(4.3%) | 1(1.1%) | 0(0%) |  |
| No treatment | 20(87.0%) | 79(86.8%) | 19(82.6%) |  |

a: 21 cases were treated with antiangiogenic therapy combined with chemotherapy, and one case was treated with antiangiogenic therapy alone; b: three cases had immunotherapy combined with chemotherapy and antiangiogenic therapy, and one case had immunotherapy combined with chemotherapy; c: radiofrequency ablation combined with radiotherapy was done in two cases, and surgery combined with radiotherapy in two cases. Abbreviations: EG, erythromycin group; ITG, intrathoracic treatment group; CG, control group; PCI, prophylactic cranial irradiation;

**Table 5 Targeted treatment group and non-targeted treatment group**

| Subgroup | Targeted treatment group | | | | Non-targeted treatment group | | | |
| --- | --- | --- | --- | --- | --- | --- | --- | --- |
| Group | EG(N=14) | ITG(N=103) | CG(N=31) | Pvalue | EG(N=23) | ITG(N=91) | CG(N=23) | P value |
| MOS(m) | 52 | 30 | 32 | 0.215 | 13 | 13 | 7 | 0.054 |
| 6-week PE-ORR | 78.6% | 73.0 | 78.6% | 0.785 | 73.7% | 47.9% | 61.1% | 0.111 |
| 6-week S-ORR | 64.3% | 63.1% | 71.0% | 0.728 | 34.8% | 35.2% | 39.1% | 0.934 |
| 12-weekPEORR | 85.7% | 74.2% | 77.8% | 0.625 | 68.8% | 65.2% | 64.7% | 0.960 |
| 12-week S-ORR | 71.4% | 54.4% | 67.7% | 0.269 | 52.2% | 36.3% | 47.8% | 0.290 |

Abbreviations: OS:overall survival；ORR:objective response rate; EG, erythromycin group; ITG, intrathoracic treatment group; CG, control group; PE-ORR: Pleural effusion objective effective rate; S-ORR: Systemic objective effective rate; ITG, intrathoracic treatment group; CG, control group;

**Table 6 ORR of erythromycin group and intrathoracic treatment group;**

| Pleural effusion | EG(N=37) | ITG(N=194) | P value |
| --- | --- | --- | --- |
| 6-week PE-ORR | 75.8% | 62.4% | 0.142 |
| 12-week PE-ORR | 76.7% | 70.5% | 0.490 |
| System |  |  |  |
| 6-week S-ORR | 44.4% | 43.3% | 0.898 |
| 12-week S-ORR | 50.0% | 36.7% | 0.170 |

The ORR was the number of CR+PR patients/total cases (excluding withdrawal cases); Abbreviations: EG, erythromycin group; ITG, intrathoracic treatment group; CG, control group; PE-ORR, pleural effusion objective remission rate; S-ORR, systemic objective remission rate;
